# Primary and Phenolic Metabolites Analyses, In Vitro Health-Relevant Bioactivity and Physical Characteristics of Purple Corn (*Zea mays* L.) Grown at Two Andean Geographical Locations

**DOI:** 10.3390/metabo11110722

**Published:** 2021-10-22

**Authors:** Lena Gálvez Ranilla, Briggite Anyela Rios-Gonzales, María Fernanda Ramírez-Pinto, Claudia Fuentealba, Romina Pedreschi, Kalidas Shetty

**Affiliations:** 1 Laboratory of Research in Food Science, Universidad Catolica de Santa Maria, Urb. San Jose s/n Umacollo, Arequipa 04001, Peru; bririos.95@gmail.com (B.A.R.-G.); mafer.rp24@gmail.com (M.F.R.-P.); 2 Escuela de Alimentos, Facultad de Ciencias Agronómicas y de los Alimentos, Pontificia Universidad Católica de Valparaíso, Av. Waddington 716, Valparaíso 2340000, Chile; claudia.fuentealba@pucv.cl; 3 Escuela de Agronomía, Facultad de Ciencias Agronómicas y de los Alimentos, Pontificia Universidad Católica de Valparaíso, Calle San Francisco s/n La Palma, Quillota 2260000, Chile; romina.pedreschi@pucv.cl; 4 Department of Plant Sciences, North Dakota State University, Fargo, ND 58108, USA; kalidas.shetty@ndsu.edu

**Keywords:** purple corn, *Zea mays* L., phenolic antioxidants, metabolomics, hyperglycemia, physical characteristics

## Abstract

Purple corn (*Zea mays* L.) is native to the Andean region, but limited research has been performed about the potential metabolic variability when grown under Andean environmental conditions. This study was aimed at evaluating the phenolic and primary polar metabolites composition of purple corn (kernels and cobs) grown at two Peruvian Andean locations (lowland and highland) using targeted UHPLC (ultra-high-performance liquid chromatography) and untargeted GC-MS (gas chromatography mass spectrometry) metabolomic platforms, respectively. Changes in the physical characteristics and the in vitro bioactivity were also determined. Purple corn from the highland zone showed higher contents of ash, crude fiber, total phenolic contents, DPPH (2,2-diphenyl-1-picrylhydrazyl) antioxidant capacity, and α-amylase inhibitory activity in kernels, whereas increased levels of flavonoids (anthocyanins and quercetin derivatives) and ABTS [2,2′-azino-bis(3-ethylbenzothiazoline-6-sulfonic acid)] antioxidant capacity were observed in cobs in comparison to lowland samples. No effect of the Andean location was found on the α-glucosidase inhibitory activity relevant for hyperglycemia management, while yield-linked physical characteristics were high in purple corn grown at the lowland zone. Polar primary metabolites related to the carbohydrate (monosaccharides, sucrose, and d-sorbitol), amino acid (valine and alanine), and tricarboxylic acid cycle (succinic, fumaric, and aconitic acid) metabolism were higher in highland purple corn (cob and kernel) likely due to abiotic stress factors from the highland environment. This study provides the foundation for further breeding improvements at Andean locations.

## 1. Introduction

Purple corn (*Zea mays* L.) is an amylaceous and purple-pigmented corn that belongs to the Peruvian race *Kculli*, which means “black” in the “Quechua” native language [1]. This race was derived from the highland pre-Columbian proto-*Kculli* race, which appeared in the final preceramic period in Peru (~4000 calibrated years before the present) and has become an important staple cereal food among indigenous communities from the Andean region in South America, including Peru, Ecuador, Bolivia, and Argentina [2,3].

In contrast to many blue and purple-pigmented corn varieties from North and Mesoamerica, Andean purple corn landraces accumulate higher concentrations of anthocyanins not only in the kernel pericarp but also in the cob and husk [4,5,6]. Previous studies have shown that whole fresh purple corn had around from 4 to 11 times higher total anthocyanin contents than blueberry fruits and that such health-relevant phenolic compounds are more concentrated in the cob than in the kernel [7]. In addition, other non-anthocyanin phenolic metabolites such as different flavonoids and phenolic acids have been confirmed in Andean purple corn [8,9,10]. Due to this high phenolic metabolite composition, purple corn has been linked to health-relevant bioactive properties. Recent in vitro and in vivo studies have reported the antioxidant, anti-hyperglycemic, anti-obesity, anti-inflammatory, and anti-cancer potential of purple corn extracts [4,11,12,13,14]. The anti-hypertensive effect of purple corn has also been demonstrated in a clinical study [15].

Based on the above bioactive potential, Peruvian purple corn germplasm has been targeted in breeding strategies to enhance the anthocyanin content in corn from other origins for potential nutritional and health applications. Paulsmeyer et al. [16] screened around 398 genetically diverse pigmented corn accessions from different origins with the objective to identify high-anthocyanin corn germplasm for breeding purposes. In this study, a Peruvian Andean purple corn accession with pigmented pericarp showed the highest anthocyanin contents, including its condensed forms [16]. Recently, Hong et al. [17] have developed a purple-pericarp super sweet sweetcorn line with anthocyanins based on purple Peruvian corn germplasm.

Although purple corn is native to the Andean region, breeding programs integrating relevant agronomic phenotypes and maximizing the phenolic bioactive composition of purple corn are still limited in Andean geographical areas. The environmental and geographical characteristics in the Andean region are quite variable, and initiatives for the enhancement of local agriculture based on purple corn production should be developed considering such heterogeneous conditions. An initial study that included the comparison of anthocyanin contents from Andean purple corn grown in different regions in Peru was reported by Jing et al. [18]. Medina-Hoyos et al. recently identified high-anthocyanin and yield of commercial purple corn varieties that better adapted to the Cajamarca region (north Andean region in Peru) [6]. Currently, purple corn is cultivated in different Peruvian regions and has been promoted as a “superfood” by the Peruvian government since 2017, which has increased its demand in the domestic and international markets [19,20]. However, the development of purple corn varieties with high quality based on their phenolic composition and adequate agronomic characteristics requires focused research considering the different environments in the Andean region.

Arequipa (south of Peru) is the second important region with high purple corn production in Peru, and this crop is currently cultivated under irrigation systems in lands located at sea level due to the high yield in terms of high ear and grain weight [21]. However, the high temperature and moisture environmental conditions common at sea level increase the incidence of pests leading to the use of pesticides. Some local farmers are currently growing purple corn at Andean altitudes (2500–3000 m above sea level, masl) in Arequipa due to the significantly less frequency of pests that allows the cultivation of purple corn naturally in organic conditions. To advance these low pest organic production strategies, the potential changes in the health-relevant metabolite composition and quality characteristics of purple corn grown at highland Andean conditions must be established.

This study was therefore aimed at comparing the metabolite composition of purple corn grown at two different locations in the Andean region of Arequipa (lowland and highland) by applying a UHPLC-DAD (ultra-high performance liquid chromatography-diode array detector) phenolic compounds targeted and GC-MS primary polar untargeted metabolites analysis of purple corn (kernels and cobs). The differences in the physical quality characteristics along with the health-relevant antioxidant and anti-hyperglycemic potential using in vitro models were also evaluated. Insights from this study would help to understand the stress and health targeted metabolites change in purple corn grown at different highland geographical locations relevant for future breeding applications advancing the improvement of local agriculture in the Andean region.

## 2. Results and Discussion

### 2.1. Physical Characteristics of Purple Corn from the Two Andean Geographical Locations

Table 1 shows the main physical characteristics evaluated in the ear, cob, and kernel from purple corn grown at lowland and highland Andean locations. The physical characteristics, such as ear weight and length along with the grain and cob color, are commonly used among local farmers for trade purposes. In this study, additional physical characteristics were considered, whereas the color was determined through the quantification of anthocyanins, as presented later in this section.

The physical characteristics of purple corn from lowland and highland zones were similar in several parameters measured in the ear (length, number of rows, number of kernels per row), cob (diameter and weight), and kernel (length and width). However, all physical characteristics related to the yield, such as ear weight, kernel weight per ear, and one-hundred-kernel weights, were higher in purple corn grown at the Andean lowland zone. Purple corn from both zones showed similar ear lengths, but samples from the lowland area showed higher ear diameter.

Chuma et al. [22] reported that several genotypes of rice grown at mid-altitude (900 masl) showed higher yields (grain yield and thousand-grain weight) than the same genotypes grown at high altitude (1600 masl). The warmer weather conditions from the mid-altitude zone might have enhanced the soil nitrogen availability and uptake by plants supporting a better grain yield [23]. In the current study, only the climatic conditions from Chuquibamba (highland) were available since the geographical localization of Iray (lowland) is close but with an average altitude difference of 700 m [24]. Maximum temperatures in the highland zone during the purple corn cultivation period ranged from 16.3 to 18.8 °C, whereas minimum values varied from 4.3 to 5.5 °C [24]. Cold stress has negative effects on the reproductive processes in grain crops and has been related to the reduction in several metabolic activities, including photosynthesis leading to the decrease in grain formation and yield [25,26,27]. It is most likely that the lowland region (Iray) had higher temperature ranges associated with the altitude difference, and this might have favored better purple corn yields; however, other factors such as the planting density, frequency of water supply, and soil fertilization have also been contributed corn yield and should be further evaluated in future studies [28].

### 2.2. Proximal Composition in Purple Corn Kernels

Purple corn, including both kernels and cobs, is commonly used in Peru for the preparation of unfermented and fermented beverages and in porridge-type desserts. However, the edible kernels could also be used in different applications such as functional food ingredients and nutraceuticals, and therefore their macronutrient composition was evaluated and is shown in Table 2.

Purple corn grown in the highland and lowland locations had similar protein, lipids, and total carbohydrate contents. However, the ash and crude fiber values were higher in purple corn kernels from the highland zone. The variability in the ash contents may be explained by differences in the soil pH (not measured in the current study), which influences the mineral solubility and consequently the plant uptake and the mineral grain composition [29]. The high crude fiber contents in kernels from the highland location might be associated with the higher levels of bound phenolic compounds also found in the same grain samples (Table 2), which are linked to insoluble carbohydrates. Phenolic compounds in grains are mainly found in a bound form (bound phenolic fraction), chemically linked with different insoluble macromolecules forming dietary fiber and significantly contributing to the total antioxidant capacity in cereal grains such as corn [30,31].

In comparison with other common cereal grains, purple corn kernels, especially from the highland zone, have higher protein and lipid contents than those reported in whole barley (8.4 and 2.0 g/100 g, respectively; [32]) and rice (5.9 and 2.0 g/100 g, respectively; [32]) whereas comparable contents have been reported in whole-grain blue corn flour (8.75 and 5.09 g/100 g of protein and lipid contents, respectively; [33]) or yellow and white corn (9.42 and 4.74 g/100 g of protein and lipid contents, respectively; [33]).

### 2.3. Purple Corn Phenolic Composition

The differences in the phenolic composition of purple corn kernel and cob from evaluated geographical zones are shown in Table 2. Considering the same location, the total phenolic (free + bound) and total anthocyanin contents are about 5–7-fold more abundant in cobs than in kernels. Independent of the purple corn structure (cob or kernel), the contribution of the free phenolic fraction to the total phenolic contents (free + bound) ranged from 77% to 86%, and the total anthocyanin concentrations in cob samples showed a high correlation with the free phenolics and total phenolic contents (*r* = 0.9888 and *r* = 0.9877, *p* < 0.05; respectively). This indicates that anthocyanins are the most important phenolic group in purple corn in contrast to what was previously reported in different non-pigmented corn (white or yellow), where dietary fiber-linked phenolic acids are the most abundant compounds [4,34]. Purple corn kernels from the highland zone showed higher total phenolic (free + bound) levels (1089.5 mg GAE, gallic acid equivalents/100 g DW, dry weight) in comparison to lowland samples (751.9 mg GAE/100 g DW). The total anthocyanin contents in purple corn kernel (327.9–414.7 mg C3G, cyanidin-3-glucoside/100 g DW) and cob (1935.3–2894.5 mg C3G/100 g DW) found in the current study are higher than ranges reported by Ranilla et al. (310 mg C3G/100 g DW, kernel) and Lao and Giusti (310–1220 mg C3G/100 g, cob), and in agreement to values reported by Ranilla et al. (76.8–510.5 mg C3G/100 g, kernel), Quispe et al. (1336–2059 mg GAE/100 g, cob) and Gorriti et al. (840.4–4798.4 mg C3G/100 g, cob) in different Peruvian purple corn samples [4,10,35,36,37]. Furthermore, lower concentrations than those determined in the current investigation have been reported by Khamphasan et al. (1176.5–2022.1 mg C3G/100 g DW, cob), Mendoza-Mendoza et al. (13.5–309 mg C3G/100 g, kernel), and Yang and Zhai (185.1 mg C3G/100 g, cob) in purple corn grown in Thailand, Mexico, and China, respectively [38,39,40].

Qualitatively, all kernel and cob samples from both locations had similar UHPLC phenolic profiles (Supplementary Figures S1–S4), but bound *p*-coumaric acid derivatives were not detected in kernel extracts. Other flavonoids besides anthocyanins, such as quercetin derivatives, were also found in both purple corn structures, but cob samples showed the highest contents (1.7–3.9 and 25.3–177.2 mg/100 g DW, for kernel and cob samples, respectively).

In relation to the effect of the geographical location, no differences were found in the flavonoid and in almost all the bound phenolic acid contents of kernels from the lowland and highland zones. However, highland kernel extracts had higher ferulic acid and total phenolic concentrations (flavonoids + phenolic acids) than lowland samples. In the case of purple corn cob, highland samples had the highest flavonoid (1160.1 and 491.7 mg/100 g DW total flavonoids for highland and lowland cob samples, respectively) and total phenolic contents (flavonoids + phenolic acids) (1896.8 and 1373.9 mg/100 g DW for highland and lowland cob samples, respectively). Major bound phenolic acids were ferulic and *p*-coumaric acids in kernel and cob samples, respectively, indicating a different phenolic composition in the dietary fiber from both purple corn structures. The phenolic acid composition of the bound phenolic fraction in purple corn cob is reported for the first time in this study and thus contributing with important information about the purple corn cob residue (commonly considered as a by-product by the industry after anthocyanin extraction), which can be used as a potential source of non-anthocyanin phenolic compounds for food ingredient applications.

Jing et al. [18] reported that purple corn cobs (cultivar PM-581) from plants grown in Cajamarca (northern Andean highland in Peru, located at 2720 masl with higher natural precipitation than in other locations) had higher anthocyanin levels (average of 549.5 mg C3G/100 g) than samples cultivated in other locations (Arequipa and Lima located at 2300 masl and at sea level, respectively, with average anthocyanin levels of 337 and 464.7 mg C3G/100 g, respectively), suggesting that climatic conditions may have an important effect on anthocyanin content in purple corn cobs. However, Medina-Hoyos et al. [6] did not find any relation between the altitude and the anthocyanin levels in cobs of purple corn cultivated in Cajamarca in environments with similar weather conditions but at different altitudes (from 2320 to 3180 masl). In the same study, the variety had a significant effect (the variety INIA 601 showed the highest content: 6.12 mg C3G/100 g DW) [6]. Similarly, Khamphasan et al. and Harakotr et al. [38,41] reported minimum or no influence of environmental conditions linked to different locations or seasons on anthocyanin contents in purple corn grown in Thailand.

Although environmental conditions from the lowland area were not available in the current study, it is most likely that stress factors such as colder temperatures and higher ultraviolet (UV) radiation commonly associated with regions located at higher altitudes may have enhanced the accumulation of ferulic acid in kernels, and flavonoids and total phenolic compounds in cob samples [42]. Ultraviolet-B radiation and cold temperatures have been identified as key environmental factors that regulate the biosynthesis of flavonoids in plants at the transcriptional level [43]. The increase in the anthocyanin contents under cold stress was observed in strawberry fruits and purple head Chinese cabbage, while the UV radiation improved the levels of flavones, flavonols, and flavanols in the pulp of peach fruits [44,45,46]. In this current research, anthocyanins and especially quercetin derivatives, which are known to contain multiple hydroxyl groups with better reactive oxygen species (ROS) scavenging capacity, may have increased in highland purple corn to counteract oxidative stress induced by UV-B and cold temperature stress factors [47]. In fact, an improvement of the DPPH and ABTS antioxidant capacity was also observed in highland kernel and cob samples (Table 3). Phenolic acids such as *p*-coumaric acid and derivatives may have been used as metabolic precursors for the biosynthesis of flavonoids, which may explain their decline in cob samples that showed the highest anthocyanin concentrations [48].

### 2.4. In Vitro Antioxidant and Anti-Hyperglycemic Activity of Purple Corn

The health-relevant functionality linked to the antioxidant and hypoglycemic potential of purple corn using in vitro assay models is shown in Table 3.

The free phenolic fractions from both purple corn structures showed the highest DPPH and ABTS free radical scavenging capacity contributing around 70–84% of the total antioxidant capacity (free + bound). In the case of the kernel, this response was significantly correlated with the total free phenolic contents (*r* = 0.9292, DPPH method), whereas a more specific correlation with the flavonoid contents (anthocyanins and quercetin derivatives) was found in cob samples (*r* = 0.8505, ABTS method). In addition, the total phenolic acids, especially the ferulic acid detected by UHPLC, highly correlated with the antioxidant capacity measured by the ABTS method (*r* = 0.8968 and *r* = 0.9494, respectively) in purple corn kernels suggesting an important contribution of hydroxycinnamic acids to the antioxidant capacity in the bound phenolic fraction. The total ABTS antioxidant capacity (free + bound) in kernels from this study (5194–5805 µmol TE/100 g DW) agreed with ranges previously reported in Peruvian purple corn accessions from the same region (Arequipa) (3091.43–6763.70 µmol TE/100 g DW) [4]. Purple corn cobs showed around 4–7 times higher antioxidant capacity values than kernel samples that were correlated to the total phenolic (free + bound) and anthocyanin contents.

Kernel and cob samples from the highland location showed significantly higher total in vitro antioxidant capacity than lowland purple corn samples (2080 and 38,424 µmol TE/100 g DW in kernel and cob extracts with the DPPH and ABTS methods, respectively). The improvement in the antioxidant capacity may be related to changes in the phenolic antioxidant composition since flavonoids with significant free radical antioxidant activity such as quercetin derivatives were more concentrated in highland kernel and cob samples, as stated previously. These variations might be associated with environmental stress factors linked to the highland location, as also was analyzed in the case of the phenolic compounds. The accumulation of flavonoids and the consequent antioxidant response has been reported in several plant species [43]. Further, the antioxidant capacity measured by the DPPH and FRAP methods increased in rice seedlings cultured under cold stress [49].

The inhibition of carbohydrate-hydrolyzing enzymes such as α-amylase and α-glucosidase at the intestinal level using oral drugs is a current therapeutic approach for preventing postprandial blood glucose increase [50]. The intake of similar food-derived natural inhibitors might be an important dietary strategy to manage hyperglycemia toward decreasing the risk of type-2 diabetes. Purple corn samples (free phenolic extracts from kernel and cobs) showed in vitro inhibitory effects against α-amylase and α-glucosidase, which suggested the relevant potential for hyperglycemia modulation (Table 3). Independent of the geographical location, all purple corn kernels and cob extracts strongly inhibited the α-glucosidase enzyme in a dose-dependent manner. However, cob samples showed higher inhibitory activities (from 83.4% to 95.0% at 0.5–1.25 mg sample) than kernel extracts (from 45.8% to 70.7% at 2.5–6.25 mg sample), whereas no significant influence of the geographical location was observed. Likely, the higher flavonoid concentrations found in cob samples partially explain this functional property, but no specific correlation was found between any phenolic compound and the α-glucosidase inhibition. Further research is needed to elucidate whether other soluble bioactive compounds not detected in the current study are involved.

The α-glucosidase inhibitory activities evaluated in the current investigation are higher than values previously reported in the free phenolic fraction from other Peruvian purple corn accessions (11.7–70.0%, 5–25 mg sample), in pigmented Chinese waxy corn (~32–36%, 1000 mg sample), and in colored Australian whole sorghum genotypes (10–15%, at 4 mg sample) [4,51,52]. In contrast, higher inhibitory activities have been reported in the free phenolic extracts from different colored quinoa varieties (~38–78%, 10–20 µg sample) [53].

In the case of the α-amylase inhibitory activities, purple corn cob extracts also showed superior values than kernels (from 8.2% to 28.9% at 10–25 mg sample and from 5.9% to 20.8% at 50–125 mg sample, respectively). However, the inhibitory ranges were moderate when compared to the α-glucosidase inhibitory activity results. This balance is beneficial for the control of hyperglycemia-relevant postprandial glucose absorption since excessive inhibition of pancreatic α-amylase may cause several side effects at the gut level [54]. In general, kernel and cob extracts from purple corn grown at the highland location showed higher α-amylase inhibitory activities, and this might be associated with flavonoids such as anthocyanins. The total free phenolic contents significantly correlated with the α-amylase inhibitory activity in the kernel (*r* = 0.9099 and *r* = 0.8355 at 50 and 125 mg of sample dose, respectively). Moreover, a high correlation between the total anthocyanin contents and the α-amylase inhibitory activity was found in cob samples (*r* = 0.8386, *r* = 0.8822, and *r* = 0.8639 at 10, 12.5, and 25 mg of sample dose, respectively). Acylated anthocyanins isolated from black carrots have shown stronger inhibition of the pancreatic α-amylase enzyme compared to anthocyanin-3-glucosides [55]. Purple corn anthocyanin composition also includes acylated forms, which could explain its α-amylase inhibitory potential [3]. In addition to the inhibition of carbohydrate-hydrolyzing enzymes, anthocyanins have been shown to prevent hyperglycemia through other different mechanisms [56].

### 2.5. Untargeted GC-MS Metabolomic Analysis of Polar Metabolite Compounds in Purple Corn and Principal Component Analysis (PCA)

**Figure 1:**
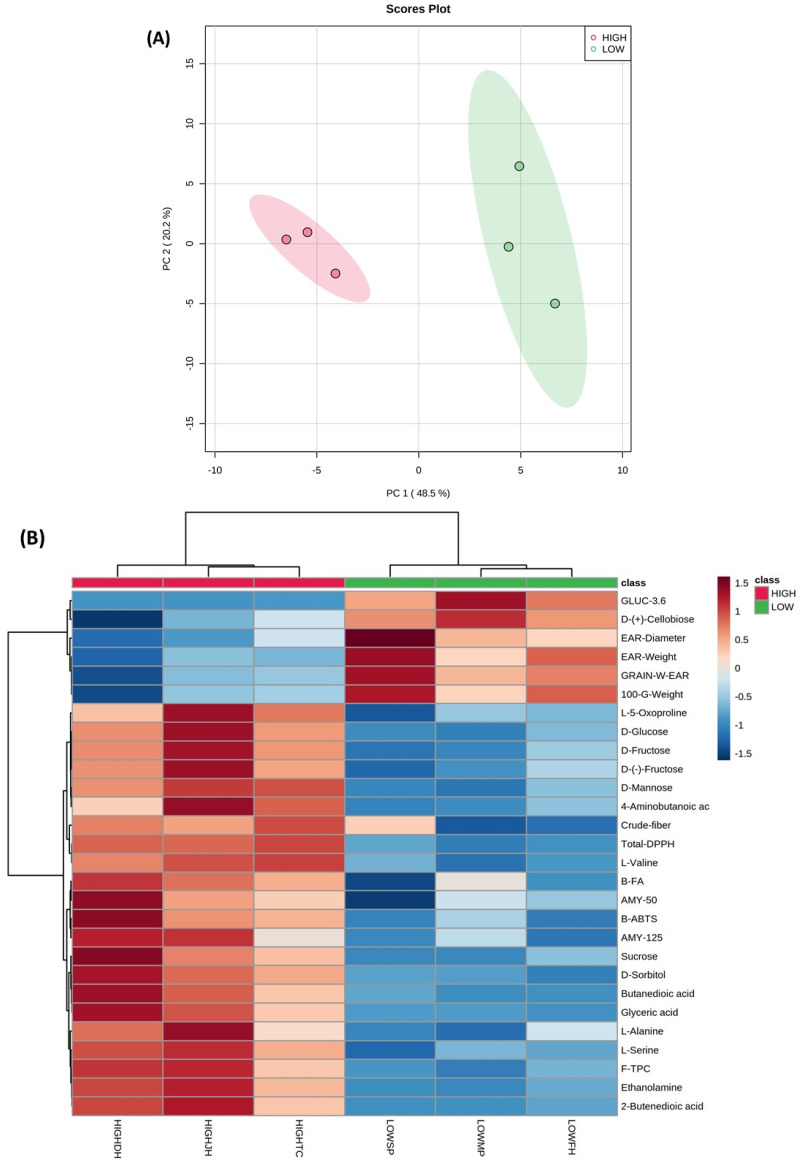
Principal component analysis (PCA) score plot (**A**) and heat map (**B**) considering significant variables from purple corn kernel samples grown at two different geographical locations (highland, HIGH and lowland, LOW). Variables in legend: GLUC-3.6, α-glucosidase inhibition (3.6 mg); GRAIN-W-EAR, kernel weight per ear; Total-DPPH, total DPPH antioxidant capacity; B-FA, bound ferulic acid; AMY-50, α-amylase inhibition (50 mg); AMY-125, α-amylase inhibition (125 mg); B-ABTS, bound ABTS antioxidant capacity; F-TPC, free total phenolic compounds.

**Figure 2:**
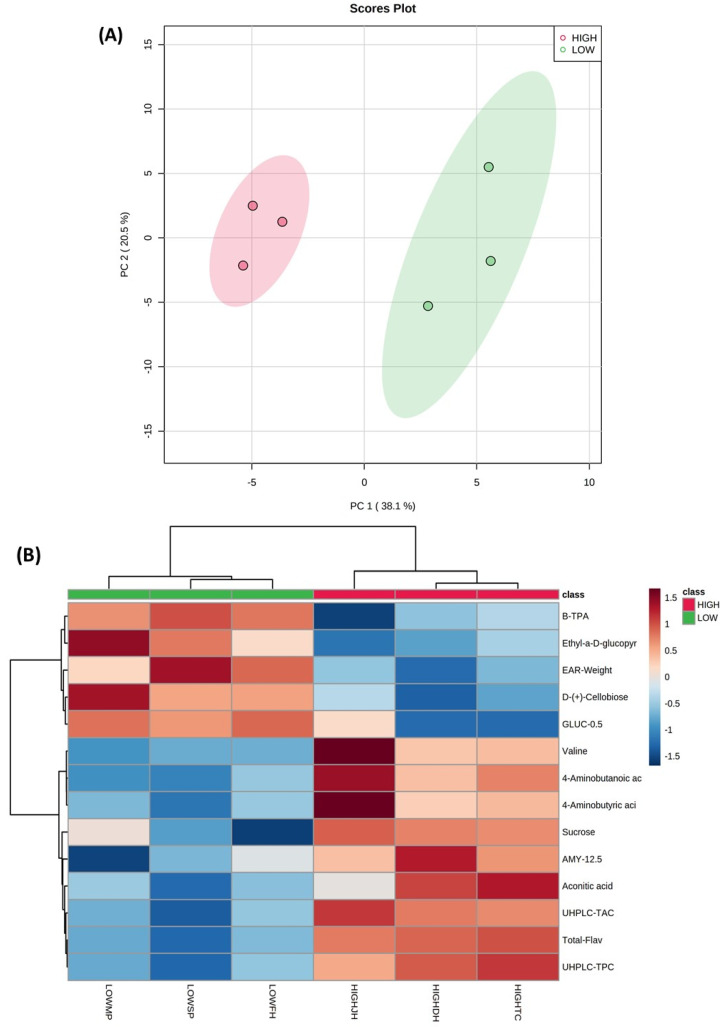
Principal component analysis (PCA) score plot (**A**) and heat map (**B**) considering significant variables from purple corn cob samples grown at two different geographical locations (highland, HIGH and lowland, LOW). Variables in legend: B-TPA, bound total phenolic acid contents; Ethyl-a-D-glucopyr, ethyl-a-D-glucopyranoside; GLUCO 0.5, α-glucosidase inhibition (0.5 mg); AMY-12.5, α-amylase inhibition (12.5 mg); AMY-10, α-amylase inhibition (10 mg); UHPLC-TAC, UHPLC total anthocyanins; Total-flav, total flavonoids; UHPLC- TPC, UHPLC total phenolic compounds

Polar metabolites including sugars, alcohols, amino acids, and organic acids were detected by GC-MS in purple corn kernel and cob samples (data not shown). A PCA was performed to evaluate the relationships among all variables (polar primary metabolites, phenolic secondary metabolites, in vitro bioactive properties, and physical characteristics) measured in kernel and cob samples from both geographical Andean locations (Figure 1 and Figure 2, respectively). The first two principal components from the PCA models explained 58.6% and 68.7% of the total variability in the case of cob and kernel data, respectively. A clear discrimination due to the geographical location (highland and lowland) was observed in both purple corn kernel and cob data sets, which was explained by 28 and 14 significant variables (*p* < 0.05), respectively (heat maps in Figure 1 and Figure 2). Mass spectra of significant polar primary metabolites detected in purple corn kernel and cob samples by CG-MS are shown in Supplementary Figures S5 and S6, respectively. Figure S7 also shows the heat map considering the top 25 *t*-test variables in the case of purple corn cob (Supplementary Material).

Kernel samples from the highland location had higher contents of sugars, including monosaccharides (d-mannose, d-fructose), sucrose, and d-sorbitol. This sugar alcohol and sucrose were also increased in highland cob samples (heat maps in Figure 2 and Figure S1). d-sorbitol is a metabolic intermediate involved in the metabolism of d-fructose, and d-mannose, which in turn are implicated in glycolysis/gluconeogenesis reactions and the nucleotide sugar metabolism relevant for carbohydrate biosynthesis [48]. Bilska-Kos et al. [57] reported the increased expression of sucrose-phosphate synthase enzyme in leaves of a chilling-sensitive maize line. This enzyme regulates the biosynthesis of sucrose from fructose-6-phosphate and uridine diphosphate glucose (UDP-glucose, a nucleotide sugar) [48]. It is most likely that d-fructose and d-mannose were used as metabolic precursors of nucleotide sugars leading to the biosynthesis of sucrose under stressed environmental conditions linked to the highland location in this current investigation. Soluble sugars, including different monosaccharides, and sucrose play a role in plant growth and adaptation to cold and stress conditions by regulating the osmotic potential of cells and stabilizing membranes [58]. The increase in sucrose along with other monosaccharides has also been reported in maize leaves and tartary buckwheat seedlings in response to cold stress [57,59].

An increase in the anthocyanin content (cyanidin-3-*O*-glucoside and cyanidin-3-*O*-rutinoside) in tartary buckwheat seedlings correlated with increases in sugars levels in response to cold, which suggested that sugars and specifically sucrose might act as signaling molecules that induce the expression of genes linked to anthocyanin biosynthesis [59,60]. This may explain the higher sucrose and total anthocyanin levels found in highland purple corn cob samples (heat map in Figure 2). In fact, significantly higher total phenolic and anthocyanin contents were found in purple corn cob than in kernels.

d-cellobiose was detected at lower levels in kernel and cob samples from the highland location when compared with samples from the lowland zone. This disaccharide may have been used as a substrate for the biosynthesis of cellulose, explaining the higher contents of crude fiber found in kernels from purple corn grown at highland conditions. The expression of the sucrose synthase enzyme involved in the biosynthesis of cellulose and starch was increased in leaves of *Miscanthus giganteus* due to cold stress [57]. In addition, methyl sugars such as the methyl-α-d-ribofuranoside may have been involved in the biosynthesis of cell wall polysaccharides in highland cob samples (Figure S1) [61].

The increase in amino acids such as valine and alanine in kernels and valine in cob samples likely indicates the provision of precursors to support the biosynthesis of multiple metabolites, including secondary metabolites such as phenolic compounds in response to highland zone linked-cold or UV radiation factors. Branched-chain amino acids such as valine have also been reported to be produced in plants as an alternative carbon source due to the increased energy demands under stress conditions [62,63]. In the current study, the levels of intermediate metabolites involved in the tricarboxylic acid (TCA) cycle were higher in highland purple corn than in lowland samples. The 2-butenedioic acid or fumaric acid and butanedioic acid or succinic acid detected in kernels, and the conjugate acid of cis-aconitate (aconitic acid) found in cob samples was increased under highland conditions. These changes in TCA cycle-derived metabolites might reflect a high mitochondrial activity with consequent generation of ATP and reducing agents as a protective mechanism to environmental abiotic stress [63]. The increase in organic acids linked to the TCA cycle has also been reported in buckwheat seedlings, and maize leaves subjected to cold treatments with variable profiles depending on the range of the temperature decrease [59,63,64].

It has been reported that levels of 4-aminobutanoic acid (γ-aminobutyric acid, GABA) were increased in the cytosol under abiotic stress conditions [65]. GABA is then transported into the mitochondria to be converted into succinate, which finally enters the TCA cycle for energy production [65]. In the current research, the increase in GABA levels in highland purple corn kernels and cob samples may have enhanced the TCA cycle activity in response to the highland Andean environment. Furthermore, other metabolites such as ethanolamine and glyceric acid were also found in higher levels in highland purple corn kernels. These metabolites may have been used as metabolic precursors for the biosynthesis of glycerolipids at the membrane level. Different changes in the membrane lipid metabolism, such as the increase in phosphatidylethanolamine and phosphatidic acid, have been observed in the roots of maize seedlings under cold stress [66].

Other variables linked to the phenolic contents, the in vitro health-relevant functionality, and yield-related physical characteristics from purple corn and cob samples have also been significantly affected by the Andean geographical location as revealed by the PCA analysis (Figure 1 and Figure 2). The multivariate analysis confirmed the differences in such variables as previously discussed. Purple corn grown at the lowland area showed higher values of ear diameter, one-hundred-kernel weight, and kernel weight per ear, indicating higher ear and grain yield than purple corn grown at the highland location. However, highland purple corn kernels correlated with higher contents of free phenolics (mainly anthocyanins), bound ferulic acid, and showed increased levels of total in vitro DPPH antioxidant capacity, bound ABTS antioxidant capacity, and α-amylase inhibition. In addition, highland cob samples correlated with higher anthocyanins, quercetin derivatives, total flavonoids, and total UHPLC phenolic contents and exhibited the highest total in vitro antioxidant capacity (DPPH method) and α-amylase inhibition.

## 3. Materials and Methods

### 3.1. Materials

The commercial purple corn variety “Canteño” was selected for the current study. This variety was cultivated from November 2017 to May 2018 in the province of Condesuyos (region of Arequipa), which is in Southern Andes in Peru. Corn plants were grown under traditional agronomic practices following organic-type cultivation (use of plow and compost for land preparation, application of natural biocides based on onion and garlic extracts, manual control of weed, and flood irrigation using natural water coming from the Andean mountains). Corn ears at commercial maturity were collected (maximum ear length, uniform grain purple color along the ear, without any visual defects) in May 2018 following the harvest period calendar used by local farmers. Ears were sampled from two different districts (Iray and Chuquibamba), which are located at different altitudinal geographical zones (Table 4). Three different farms or lands were selected per district, and purple corn ears collected from each farm/land were considered as biological replicates (*n* = 3). Around 2 kg of mature purple corn ears (5–7 units with husk) were randomly collected from each farm, transported at 5 °C to the laboratory, the husks were eliminated, and finally, ears were dried in an air forced oven at 42 °C for three days. This procedure mimicked common postharvest practices in the field where the de-husked entire purple corn ears are directly exposed to the sun for several days. A controlled drying process of purple corn ears was followed for the current study as described above. The dried purple corn ears were first analyzed based on their physical characteristics. Then, the kernels and cobs were separately pooled (per replicate), milled in a coffee grinder to 500 µm, and stored as powdered flour at −20 °C until analysis. A subsample of 50 g kernel and cob flour was used for the polar metabolite analysis and in vitro functional analyses.

### 3.2. Enzymes and Reagents

Baker yeast *α*-glucosidase (EC 3.2.1.20, G5003-100 UN catalog number, ≥10 units/mg protein), *α*-amylase (EC 3.2.1.1, A3176-10 MU catalog number, ≥10 units/mg solid) were purchased from Sigma Chemical Co. (St. Louis, MO, USA). Phenolic standards (*p*-coumaric acid, ferulic acid, quercetin, cyanidin chloride, gallic acid), the Folin–Ciocalteu reagent, methoxyamine hydrochloride, phenyl-β-d-glucopyranoside, pyridine and N,O-bis(trimethylsilyl)trifluoro-acetamide (BSTFA) were purchased from Sigma Chemical Co.

### 3.3. Measurement of Physical Characteristics

Physical characteristics in the dried ears (length, diameter, weight, number of rows, number of kernels per row, kernel weight per ear, % kernel weight per ear), cob (diameter and weight), and kernels (weight of 100 kernels, length, and width) were measured according to the International Board for Plant Genetic Resources [67].

### 3.4. Chemical Proximal Composition of Purple Corn Kernels

The purple corn kernels (edible section of purple corn traditionally used as flour for human consumption) were assayed for contents of moisture (gravimetric method at 105 °C until constant weight), lipids (with organic solvent), total protein (Kjelhdal method, using the nitrogen-to-protein conversion factor of 6.25), ash (incineration in a muffle furnace at 500 °C), and crude fiber (gravimetric method after chemical digestion) [68]. Total carbohydrates were determined by the difference of these contents.

### 3.5. Extraction of the Free and Bound Phenolic Fractions from Purple Corn Kernel and Cob Samples

Free and bound phenolic fractions were extracted according to the method reported by Ranilla et al. [4,10]. The free phenolic compounds in the kernel (5 g) and cob (1 g) were extracted using 20 mL of 0.1% HCl methanol/acetone/water (45:45:10, *v/v/v*) under agitation in an orbital shaker at 200 rpm for 63 min at ambient temperature and in the absence of light. A second extraction was applied on the residue under the same solvent conditions, and both recovered supernatants (after centrifugation at 2665× *g* for 15 min) were vacuum-evaporated to dryness at 45 °C and reconstituted in 10 mL with Milli-Q water.

The bound phenolic compounds were released by alkaline hydrolysis. An amount of 0.5 g and 0.1 g of kernel and cob samples, respectively, were mixed with 2 mL of 20 mL of 0.1% HCl methanol/acetone/water (45:45:10, *v/v/v*), and the free phenolic compounds were extracted under the same experimental conditions as stated above. Supernatants were discarded, and the residue was mixed with 20 mL of 3 N NaOH, and the hydrolysis was performed under agitation (200 rpm) for 88 min at room temperature. The mixture was acidified with HCl to pH 2.5, and the released phenolic compounds were extracted six times with 10 mL of ethyl acetate. The ethyl acetate fractions were recovered, mixed, vacuum-evaporated to dryness at 45 °C, and reconstituted in 5 mL of Milli-Q water [4,10].

Kernel and cob aqueous free and bound phenolic extracts were corrected to a pH of 7.0 only for enzymatic assays and kept at −20 °C.

### 3.6. Extraction of Anthocyanin Compounds from Purple Corn Kernel and Cob Samples

The methods of Lao and Giusti, Jing et al., and Jing and Giusti were compared for evaluating the best conditions for anthocyanin extraction in purple corn (kernel and cob) samples [5,18,69]. The method of Lao and Giusti, which is based on the use of 0.01% 6 N HCl (*v/v*) acidified 50% aqueous ethanol, was selected since it provided the highest total monomeric anthocyanin contents when compared with the other two methods. Final extracts were made up to 50 and 10 mL with 0.01% 6 N HCl acidified water for cob and grain samples, respectively, and stored at −20 °C until analysis [5]. These extracts were used for the determination of the total monomeric anthocyanin contents and UHPLC liquid chromatographic assays.

### 3.7. Total Phenolic and Total Monomeric Anthocyanin Contents

The total phenolic contents were assayed as both the free and bound phenolic extracts from kernel and cob samples according to the Folin–Ciocalteu method [70]. Results were expressed as mg of gallic acid equivalents (GAE) per 100 g (dry weight basis, DW) using a calibration curve of gallic acid standard (Y = 30.893X + 0.0228, *r*^2^ = 0.9980)

The total monomeric anthocyanin contents were determined by the pH differential method by diluting the kernel and cob extracts with 0.025 M potassium chloride (pH 1.0) and 0.4 M sodium acetate (pH 4.5) buffers. The absorbances of each diluted extract were measured at 511 and 700 nm using a UV-1700 UV-visible spectrophotometer (Shimadzu Corporation, Tokyo, Japan). The calculation of the total monomeric anthocyanin contents was performed using the following equation:

C=(A∗MW∗DF∗1000)/(ε∗1)

where *C* is the concentration of anthocyanins in mg/L, *A* is the sample absorbance, and *DF* is the dilution factor. *MW* and *ε* correspond to the molecular weight and absorptivity (449.2 g/mol and 26,900 L/mol*cm, respectively) of cyanidin-3-glucoside. Results were expressed as mg of cyanidin-3-glucoside (mg C3G) per 100 g (dry weight basis, DW) [71].

### 3.8. Analysis of Phenolic Profiles by Ultra-High Performance Liquid Chromatography (UHPLC)

Kernel and cob anthocyanin extracts in 0.01% 6N HCl acidified water were purified using a Strata solid phase extraction (SPE) C18-E column (6 mL, 500 mg, 55 µm, 70 A°) (Phenomenex Inc., Torrance, CA, USA) following the procedure of Rodriguez-Saona and Wrolstad [72]. Final purified extracts were made up to a known volume with 0.01% HCl acidified water. The anthocyanin purified extracts and the bound phenolic fractions from kernel and cob samples were filtered with a polyvinyldiene difluoride filter (PVDF, 0.22 µm) for UHPLC analysis.

A volume of 10 and 5 µL of the purified anthocyanin and bound phenolic extracts (kernel and cob), respectively, were injected in an Ultimate 3000 RS UHPLC system (Thermo Fisher Scientific, Waltham, MA, USA) with a Vanquish diode array detector, autosampler and quaternary pump, and controlled by the Chromeleon SR4 software version 7.2 (Thermo Fisher Scientific). The column used was 100 × 2.1 mm i.d., 1.7 µm Kinetex C18 (Phenomenex Inc., Torrance, CA., USA) protected with a 5 × 2.1 mm i.d., 1.7 µm Kinetex C18 guard column (Phenomenex Inc., Torrance, CA., USA). The elution solvents were as follows: A, 0.1% formic acid (pH 2.5), and B, acetonitrile. Solvent gradient and chromatographic conditions were the same as those used by Vargas-Yana et al. [73]. The initial conditions were 98% (A) and 2% (B), and the solvent (B) changed as follows: 2% for 2 min, then changed to 15% in 1 min and to 45% in the next 6 min, then increased to 98% over 1 min and maintained for 3 min, and set to initial conditions in 4 min with an equilibration period of 3 min (total run time of 20 min). The eluates were monitored from 200 to 600 nm, the peak identification was performed by comparison of retention times and diode array spectral characteristics with the external standards and the library spectra, and the quantification of phenolic compounds was based on calibration curves built with pure phenolic standards (*r*^2^ = 0.9990). Total anthocyanins were quantified at 525 nm as cyanidin chloride (Y = 3,123,271X − 611.9), whereas quercetin derivatives were quantified at 360 nm and expressed as quercetin aglycone (Y = 2,482,348X − 320.0). The phenolic acids such as *p*-coumaric and ferulic acid were quantified at 320 nm and expressed as their respective aglycone standards (Y = 3,680,745X − 1502 and Y = 2,942,673X + 411 for *p*-coumaric and ferulic acid, respectively). The ferulic and *p*-coumaric acid derivatives (with similar spectral characteristics to ferulic and *p*-coumaric acids, but with different retention times) were quantified as ferulic and *p*-coumaric acids, respectively, using their corresponding calibration curves (shown above). Results were presented as mg per 100 g sample DW.

### 3.9. 2,2-Diphenyl-1-picrylhydrazyl Radical (DPPH^·^) and 2-2′-Azino-bis(3-Ethylbenzothiazoline-6-Sulfonic Acid) Radical Cation (ABTS^·+^) Inhibition Antioxidant Capacity

The DPPH and ABTS inhibition antioxidant assays were determined in the free and bound phenolic fractions (kernel and cob samples) according to Perez-Jiménez and Saura-Calixto [74]. Results were expressed as µmol of Trolox equivalents (TE) per 100 g DW based on a curve of Trolox as a standard (50–600 µM Trolox) for both antioxidant assays (Y = 0.1162X − 2.989, *r*^2^ = 0.9990 and Y = 0.149X − 2.780, *r*^2^ = 0.9980 corresponding to the ABTS and DPPH antioxidant assays, respectively).

### 3.10. Inhibitory Activity against α-Amylase and α-Glucosidase Enzymes

The anti-hyperglycemic-relevant *α*-amylase and α-glucosidase inhibitory activities of kernel and cob extracts (free and bound phenolic fractions) were assayed according to González-Muñoz et al. [34]. The results were expressed as percentage of inhibition at different sample amounts.

### 3.11. Gas Chromatography Mass Spectrometry (GC-MS) Untargeted Metabolome Analysis of Polar Compounds

The extraction and derivatization conditions for the analysis of polar metabolites by GC-MS were performed as described by Fuentealba et al. [75]. The powdered sample (20 mg) was mixed with 500 µL of cold methanol and 20 µL of 2910 ng/µL phenyl-β-d-glucopyranoside (internal standard). Methoximation was performed by adding 120 µL of 20 mg/mL of methoxyamine hydrochloride and incubated at 70 °C for 15 min with shaking. Then, 120 µL de BSTFA were added and incubated at 37 °C for 30 min with shaking. The samples (1 µL) were injected in an Agilent 7890B gas chromatography system with a 5977A single quadrupole MS, an electron impact ionization source, a PAL3 autosampler, and an HP-5ms Ultra Inert column (30 m × 0.25 mm × 0.25 µm) (Agilent Technologies, Santa Clara, CA, USA). The injector, interface, MS ion source, and quadrupole temperatures were 220, 280, 230, and 150 °C, respectively. Mass spectra in the 50–600 *m*/*z* range were recorded at a scanning speed of 2.66 scan cycles per second. Two GC-MS methods were performed; the first one included an injection with a split ratio of 1:150, and an oven temperature starting at 120 °C (for 1 min), increasing to 300 °C at 10 °C/min, and then held for 6 min. The second method used a splitless injection mode, and the oven temperature was programmed to start at 50 °C (for 1 min), increase to 310 °C at 10 °C/min and then hold for 13 min. For both methods, helium (Indura, Santiago, Chile) was used as a carrier gas with a constant flow of 1 mL/min.

The deconvolution and identification of peaks were performed by comparing the retention times and mass spectra to a home-built library of commercial standards and NIST14 library using Mass Hunter Quantitative software (Agilent Technologies, Santa Clara, CA, USA). Results were expressed as a relative response of each detected compound based on its respective sample weight, the internal standard, and a quality control (QC) sample representative of all samples.

### 3.12. Statistical Analysis

All results were expressed as means ± standard deviation. The data were analyzed by a *t*-test comparison of independent means (α = 0.05) using the Statgraphics Centurion XVI (StatPoint Inc., Rockville, MD, USA) software. A pooled sample (cob or kernels) per land was considered as a biological replicate (*n* = 3) at the two Andean geographical localizations (lowland and highland) (Table 4).

The unsupervised multivariate analysis, principal component analysis (PCA), was performed using the software Metaboanalyst version 5.0 (https://www.metaboanalyst.ca/, accessed on 19 July 2021). Results from the metabolite profile, the in vitro functionality, and physical characteristics of both categories (lowland and highland) were evaluated simultaneously for this PCA analysis. In addition, heat maps or cluster analyses based on Euclidean distance and the Ward algorithm were built in Metaboanalyst, considering only the significant metabolites revealed by a *t*-test (*p* < 0.05).

## 4. Conclusions

Purple corn grown at two Peruvian Andean locations (lowland and highland) showed differences in the primary and secondary metabolite composition, the in vitro health-relevant functional quality, and some physical characteristics. Purple corn kernels from plants cultivated at the highland zone had higher contents of ash, crude fiber, total phenolic contents, bound ferulic acid, DPPH total antioxidant capacity, and α-amylase inhibitory activities, whereas cobs showed increased levels of flavonoids (anthocyanins and quercetin derivatives), and ABTS total antioxidant capacity than lowland samples. Purple corn (kernels and cob) showed a strong α-glucosidase inhibitory activity with no effect on the Andean location. However, higher yield-linked physical characteristics were observed in purple corn grown at the lowland zone. This is the first metabolomic study applied in purple corn grown in Andean locations and shows that polar primary metabolites related to the carbohydrate (monosaccharides, sucrose, and d-sorbitol), amino acid (valine and alanine), and tricarboxylic acid cycle (succinic, fumaric, and aconitic acid) metabolism were up-regulated in highland purple corn (cob and kernel). Likely abiotic stress factors associated with highland Andean environments may explain such differences, but more research is needed to understand the metabolic differences in purple corn adapted to the environmental conditions of the Andean region. These preliminary metabolomic and health-relevant functional results are part of the evolving biochemical and molecular foundation for further advancing quality parameters for breeding strategies targeting a better balance among relevant phenotypes such as the yield, the phenolic antioxidant contents, and the health-relevant bioactivity in purple corn cultivated in the Andean region.

## Figures and Tables

**Table 1:** Physical characteristics of purple corn from lowland and highland geographical zones in Peru.

Corn Part	Characteristics	Lowland	Highland
Ear	Length (cm)	14.1 ± 1.0 ^a^	14.2 ± 0.5 ^a^
	Diameter (cm)	4.4 ± 0.2 ^a^	3.9 ± 0.1 ^b^
	Weight (g)	124.7 ± 14.3 ^a^	84.9 ± 9.2 ^b^
	Number of rows	11.0 ± 0.4 ^a^	10 ± 0.2 ^a^
	Number of kernels per row	25.0 ± 0.5 ^a^	25.0 ± 1.5 ^a^
	kernel weight per ear (g)	92.2 ± 6.9 ^a^	67.2 ± 7.3 ^b^
	% kernel weight per ear	75.7 ± 0.0 ^a^	78.4 ± 0.0 ^a^
Cob	Diameter (cm)	2.4 ± 0.2 ^a^	2.4 ± 0.1 ^a^
	Weight (g)	16.2 ± 0.5 ^a^	17.8 ± 2.5 ^a^
Kernel	100-kernel weight (g)	42.7 ± 4.7 ^a^	28.7 ± 4.8 ^b^
	Length (cm)	1.2 ± 0.1 ^a^	1.0 ± 0.1 ^a^
	Width (cm)	1.0 ± 0.1 ^a^	0.9 ± 0.0 ^a^

Different letters within the same row indicate significant statistical differences (*p* < 0.05).

**Table 2:** Proximal composition and phenolic compounds in purple corn kernel and cob from lowland and highland geographical zones in Peru.

Analyses-	Kernel		Cob	
	Lowland	Highland	Lowland	Highland
Proximal Composition				
(g/100 g)				
Moisture	10.5 ±1.4 ^a^	9.3 ± 0.6 ^a^	--	--
Lipids	4.6 ± 0.6 ^a^	4.1 ± 0.4 ^a^	--	--
Protein	8.2 ± 0.4 ^a^	8.9 ± 0.8 ^a^	--	--
Ash	1.4 ± 0.1 ^b^	1.8 ± 0.1 ^a^	--	--
Crude fiber	1.5 ± 0.6 ^b^	2.6 ± 0.2 ^a^	--	--
Total carbohydrates	75.3 ± 1.9 ^a^	75.9 ± 1.4 ^a^	--	--
Phenolic Compounds				
(mg GAE/100 g DW)				
Free	586 ± 35 ^b^	889 ± 84 ^a^	2962 ± 1108 ^a^	4425 ± 427 ^a^
Bound	166 ± 34 ^a^	201 ± 9 ^a^	880 ± 50 ^a^	694 ± 32 ^b^
Total	752 ± 30 ^b^	1090 ± 92 ^a^	3842 ± 1090 ^a^	5118 ± 428 ^a^
Total Monomeric Anthocyanins (mg C3G/100 g DW)	328 ± 53 ^a^	415 ± 59 ^a^	1935 ± 692 ^a^	2894 ± 350 ^a^
UHPLC Phenolic Profile				
(mg/100 g DW)				
Total anthocyanins ^2^	53.5 ± 5.8 ^a^	64.4 ± 10.9 ^a^	466 ± 127 ^b^	983 ± 68 ^a^
Quercetin derivatives ^3^	1.7 ± 1.5 ^a^	3.9 ± 1.3 ^a^	25.3 ± 1.8 ^b^	177 ± 109 ^a^
Total flavonoids	55.2 ± 6.8 ^a^	68.3 ± 12.3 ^a^	492 ± 127 ^b^	1160 ± 41 ^a^
Bound *p*-coumaric acid	23.4 ± 5.7 ^a^	19.5 ± 1.0 ^a^	476 ± 8 ^a^	391 ± 65 ^a^
Bound *p*-coumaric acid derivatives ^4^	ND ^1^	ND ^1^	11.8 ± 1.3 ^a^	11.6 ± 1.0 ^a^
Bound ferulic acid	108 ± 21 ^b^	153 ± 9 ^a^	380 ± 20 ^a^	322 ± 43 ^a^
Bound ferulic acid derivatives ^5^	17.4 ± 2.4 ^a^	19.5 ± 1.7 ^a^	14.1 ± 2.9 ^a^	13.1 ± 1.8 ^a^
Total bound phenolic acids	149 ± 29 ^a^	192 ± 10 ^a^	882 ± 15 ^a^	737 ± 56 ^b^
Total phenolic compounds (flavonoids + phenolic acids)	204 ± 29 ^b^	261 ± 7 ^a^	1374 ± 115 ^b^	1897 ± 96 ^a^

Different letters in a row within the same purple corn structure (kernel or cob) indicate significant statistical differences (*p* < 0.05). ^1^ not detected. ^2^ expressed as cyanidin chloride. ^3^ expressed as quercetin aglycon. ^4^ expressed as *p*-coumaric acid. ^5^ expressed as ferulic acid.

**Table 3:** In vitro antioxidant capacity, α-amylase, and α-glucosidase inhibition in purple corn kernel and cob from lowland and highland geographical zones in Peru.

Analyses-	Kernel		Cob	
	Lowland	Highland	Lowland	Highland
DPPH Antioxidant Assay				
(µmol TE/100 g DW)				
Free	957 ± 21 ^b^	1759 ± 36 ^a^	5271 ± 1731 ^a^	7380 ± 595 ^a^
Bound	290 ± 48 ^a^	322 ± 9 ^a^	2003 ± 374 ^a^	2390 ± 56 ^a^
Total	1247 ± 69 ^b^	2080 ± 41 ^a^	7274 ± 1561 ^a^	9770 ± 610 ^a^
ABTS Antioxidant Assay				
(µmol TE/100 g DW)				
Free	3746 ± 188 ^a^	4273 ± 540 ^a^	21,718 ± 3113 ^b^	28,391 ± 1771 ^a^
Bound	1448 ± 19 ^b^	1532 ± 26 ^a^	9354 ± 548 ^a^	10,033 ± 606 ^a^
Total	5194 ± 183 ^a^	5805 ± 516 ^a^	31,072 ± 3190 ^b^	38,424 ± 1263 ^a^
α-Amylase Inhibition (%)				
50 mg (kernel), 10 mg (cob)	5.9 ± 2.4 ^b^	11.0 ± 2.0 ^a^	8.2 ± 1.5 ^a^	15.6 ± 5.2 ^a^
62 mg (kernel), 12.5 mg (cob)	9.2 ± 4.4 ^a^	14.2 ± 2.5 ^a^	9.8 ± 2.6 ^b^	19.9 ± 3.9 ^a^
125 mg (kernel), 25 mg (cob)	14.9 ± 1.7 ^b^	20.8 ± 2.5 ^a^	17.7 ± 5.1 ^a^	28.9 ± 11.5 ^a^
α-Glucosidase Inhibition (%)				
2.5 mg (kernel), 0.5 mg (cob)	49.9 ± 13.2 ^a^	45.8 ± 1.6 ^a^	89.4 ± 5.5 ^a^	83.4 ± 15.9 ^a^
3.6 mg (kernel), 0.7 mg (cob)	58.9 ± 12.8 ^a^	54.9 ± 3.9 ^a^	91.8 ± 4.7 ^a^	88.5 ± 11.1 ^a^
6.25 mg (kernel), 1.25 mg (cob)	70.7 ± 11.9 ^a^	67.1 ± 2.8 ^a^	95.0 ± 4.0 ^a^	92.5 ± 7.9 ^a^

Different letters in a row within the same purple corn structure (kernel or cob) indicate significant statistical differences (*p* < 0.05).

**Table 4:** Collected purple corn samples from lowland and highland geographical zones in Peru.

District *	Name of the Land	Altitude (m)	Latitude and Longitude
Iray (Lowland)	Huichara	2249	15°50′34.2″ S 72°38′9.3″ W
	Puyara	2337	15°51′54.7″ S 72°37′7.4″ W
	Pacaichacra	1712	15°53′43.2″ S 72°34′10″ W
Chuquibamba (Highland)	Chiringay	2896	15°48′59.6″ S 72°38′33.3″ W
	Huayra A	2768	15°50′20.7″ S 72°38′33.4″ W
	Huayra B	2759	15°50′53.5″ S 72°38′33.6″ W

* Both districts are located within the province of Condesuyos in the Arequipa region, Peru.

## Data Availability

The data presented in this study are available in the article.

## Supplementary Materials

The following are available online at https://www.mdpi.com/article/10.3390/metabo11110722/s1, Figure S1: UHPLC chromatograms of purple corn kernel sample from the lowland geographical location, (A): anthocyanin free fraction at 525 nm, (B): bound phenolic fraction at 320 nm. Figure S2: UHPLC chromatograms of purple corn kernel sample from the highland geographical location, (A): anthocyanin free fraction at 525 nm, (B): bound phenolic fraction at 320 nm, Figure S3: UHPLC chromatograms of purple corn cob sample from the lowland geographical location, (A): anthocyanin free fraction at 525 nm, (B): bound phenolic fraction at 320 nm, Figure S4: UHPLC chromatograms of purple corn cob sample from the highland geographical location. (A): anthocyanin free fraction at 525 nm, (B): bound phenolic fraction at 320 nm, Figure S5: Mass spectra of significant polar primary metabolites detected in purple corn kernels by CG-MS., Figure S6: Mass spectra of significant polar primary metabolites detected in purple corn cobs by CG-MS, Figure S7: heat map considering the top 25 *t*-test variables in case of purple corn cob samples grown at two different geographical locations (highland, HIGH and lowland, LOW).
