# Supplementary material for: Primary and Phenolic Metabolites Analyses, In Vitro Health-Relevant Bioactivity and Physical Characteristics of Purple Corn (Zea mays L.) Grown at Two Andean Geographical Locations
Source: Metabolites. 2021 Oct 22;11(11):722. doi: 10.3390/metabo11110722 (PMC8625611; doi:10.3390/metabo11110722)
Supplement: Supplementary file 1 [file metabolites-11-00722-s001.zip › metabolites-1361149-supplementary.pdf]

Supplementary material

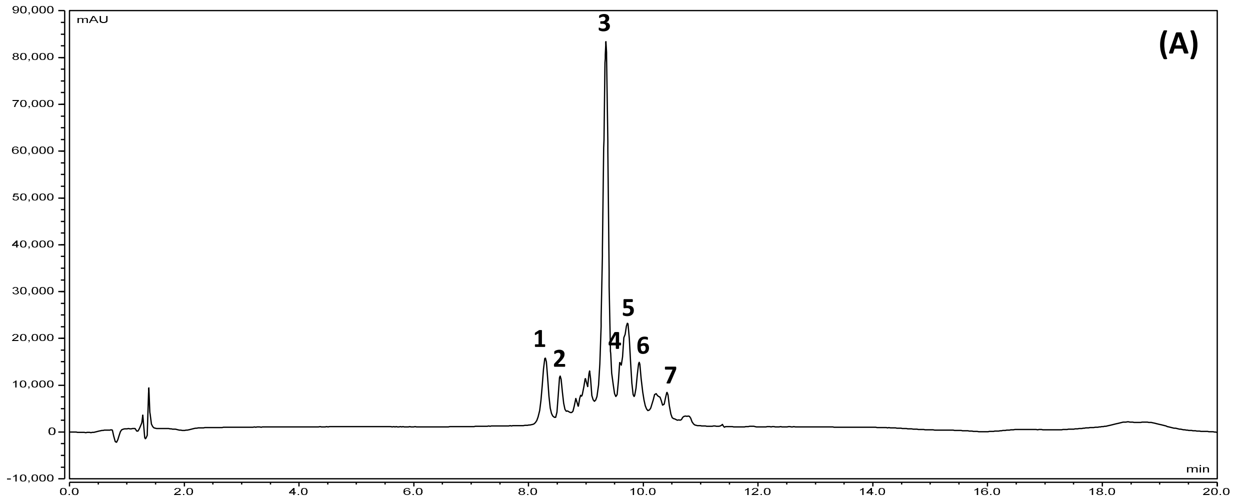

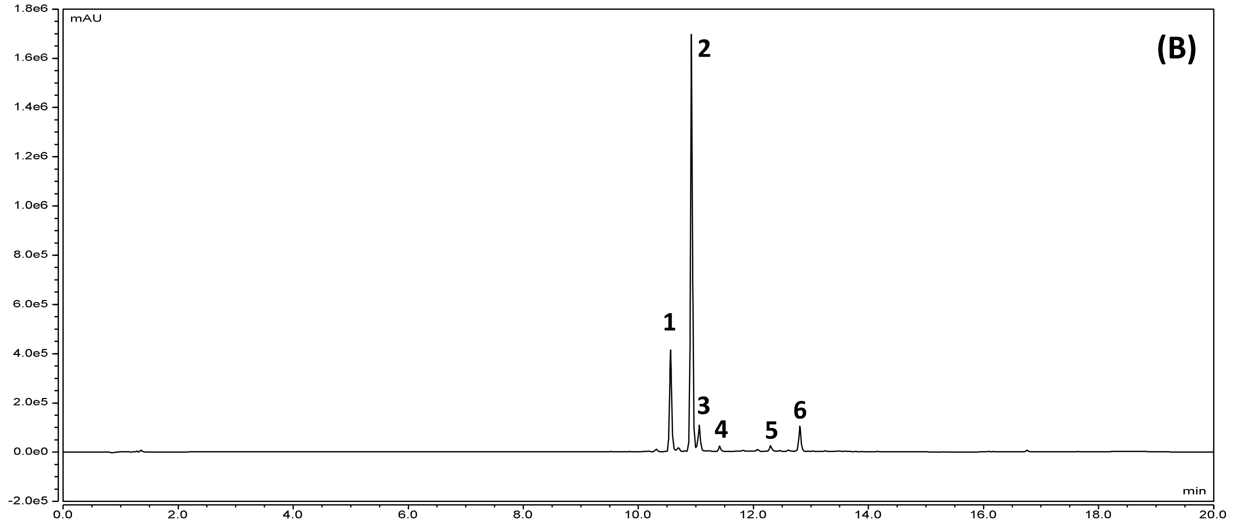

Supplementary Figure S1. UHPLC chromatograms of purple corn kernel sample from the lowland geographical location. (A): anthocyanin free fraction at 525 nm, (B): bound phenolic fraction at 320 nm.

Phenolic fraction	Peak number	Retention time (min)	λmax (nm)	Compound
Anthocyanin free fraction at 525 nm	1	8.2920	284.59; 532.85	Anthocyanin
	2	8.5520	283.14; 523.25	Anthocyanin
	3	9.3480	280.46; 515.85	Anthocyanin
	4	9.6580	281.51; 516.50	Anthocyanin
	5	9.7250	279.40; 517.86	Anthocyanin
	6	9.9300	281.42; 519.37	Anthocyanin
	7	10.415	281.32; 522.20	Anthocyanin
Bound phenolic fraction at 320 nm	1	10.560	309.72	*p*-coumaric acid
	2	10.923	323.18	Ferulic acid
	3	11.058	317.43	Ferulic acid derivative
	4	11.412	323.29	Ferulic acid derivative
	5	12.297	323.89	Ferulic acid derivative
	6	12.805	325.05	Ferulic acid derivative

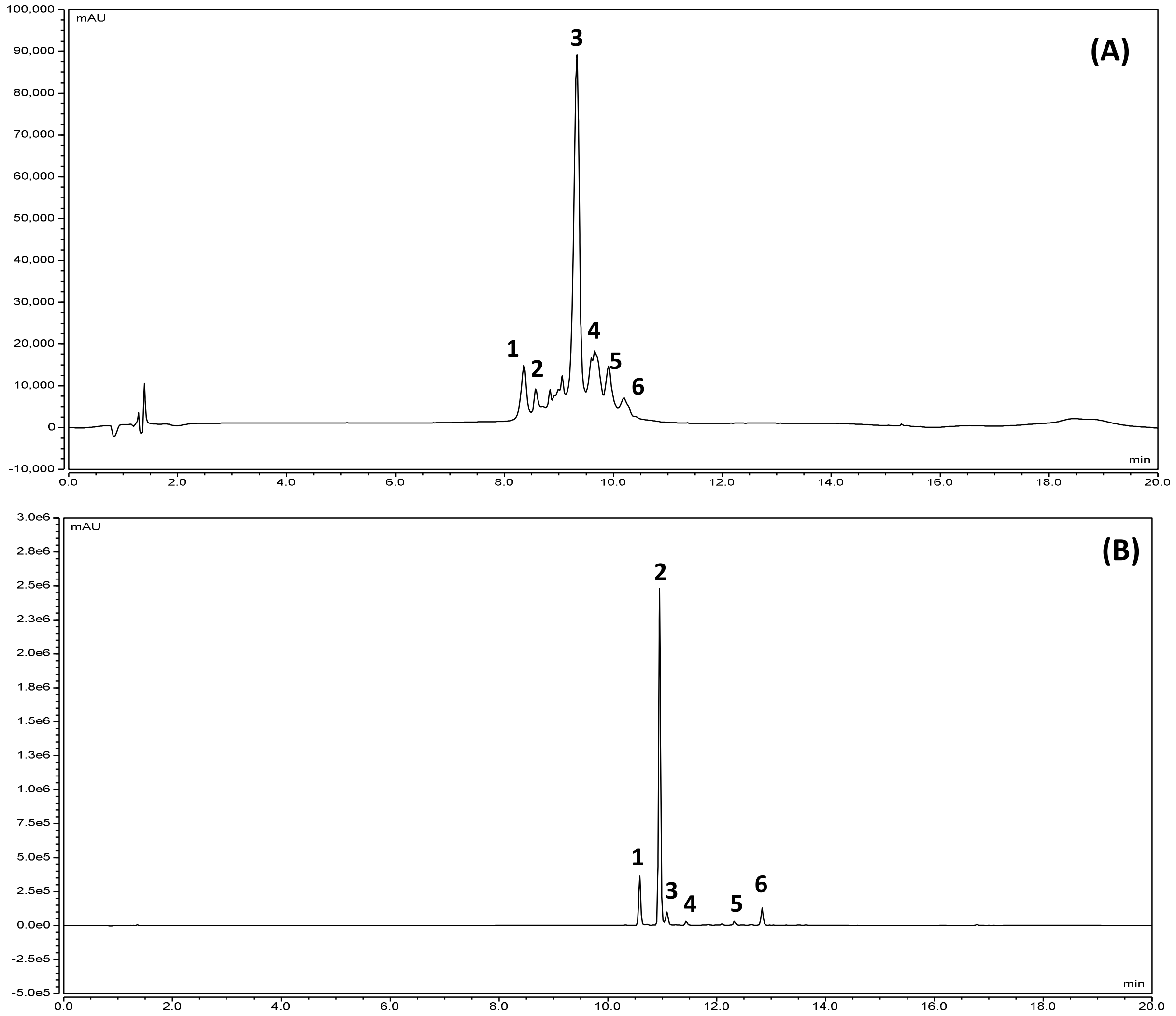

Supplementary Figure S2. UHPLC chromatograms of purple corn kernel sample from the highland geographical location. (A): anthocyanin free fraction at 525 nm, (B): bound phenolic fraction at 320 nm.

Phenolic fraction	Peak number	Retention time (min)	λmax (nm)	Compound
Anthocyanin free fraction at 525 nm	1	8.3580	284.20; 529.86	Anthocyanin
	2	8.5730	282.81; 522.58	Anthocyanin
	3	9.3300	280.67; 515.79	Anthocyanin
	4	9.7230	279.07; 517.56	Anthocyanin
	5	9.9150	281.47; 518.84	Anthocyanin
	6	10.2870	281.72; 517.99	Anthocyanin
Bound phenolic fraction at 320 nm	1	10.587	309.70	*p*-coumaric acid
	2	10.952	322.84	Ferulic acid
	3	11.088	317.56	Ferulic acid derivative
	4	11.442	323.61	Ferulic acid derivative
	5	12.327	323.92	Ferulic acid derivative
	6	12.835	324.96	Ferulic acid derivative

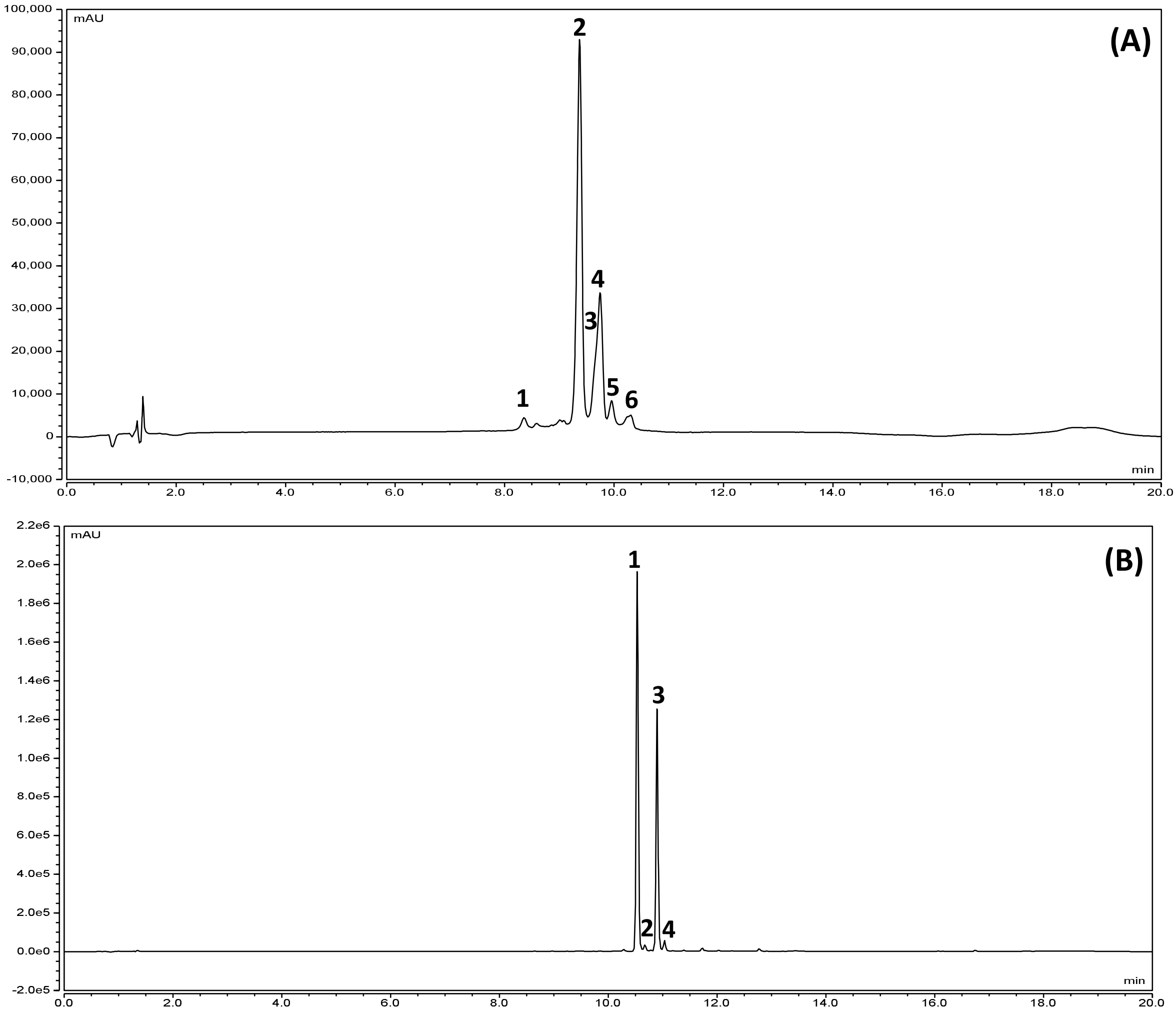

Supplementary Figure S3. UHPLC chromatograms of purple corn cob sample from the lowland geographical location. (A): anthocyanin free fraction at 525 nm, (B): bound phenolic fraction at 320 nm.

Phenolic fraction	Peak number	Retention time (min)	λmax (nm)	Compound
Anthocyanin free fraction at 525 nm	1	8.3620	285.06; 537.05	Anthocyanin
	2	9.3730	280.35; 515.64	Anthocyanin
	3	9.6370	278.83; 506.51	Anthocyanin
	4	9.7470	279.80; 516.31	Anthocyanin
	5	9.9570	281.29; 517.41	Anthocyanin
	6	10.31	283.50; 519.06	Anthocyanin
Bound phenolic fraction at 320 nm	1	10.533	309.99	*p*-coumaric acid
	2	10.673	300.90	*p*-coumaric acid derivative
	3	10.898	323.20	Ferulic acid
	4	11.037	317.63	Ferulic acid derivative

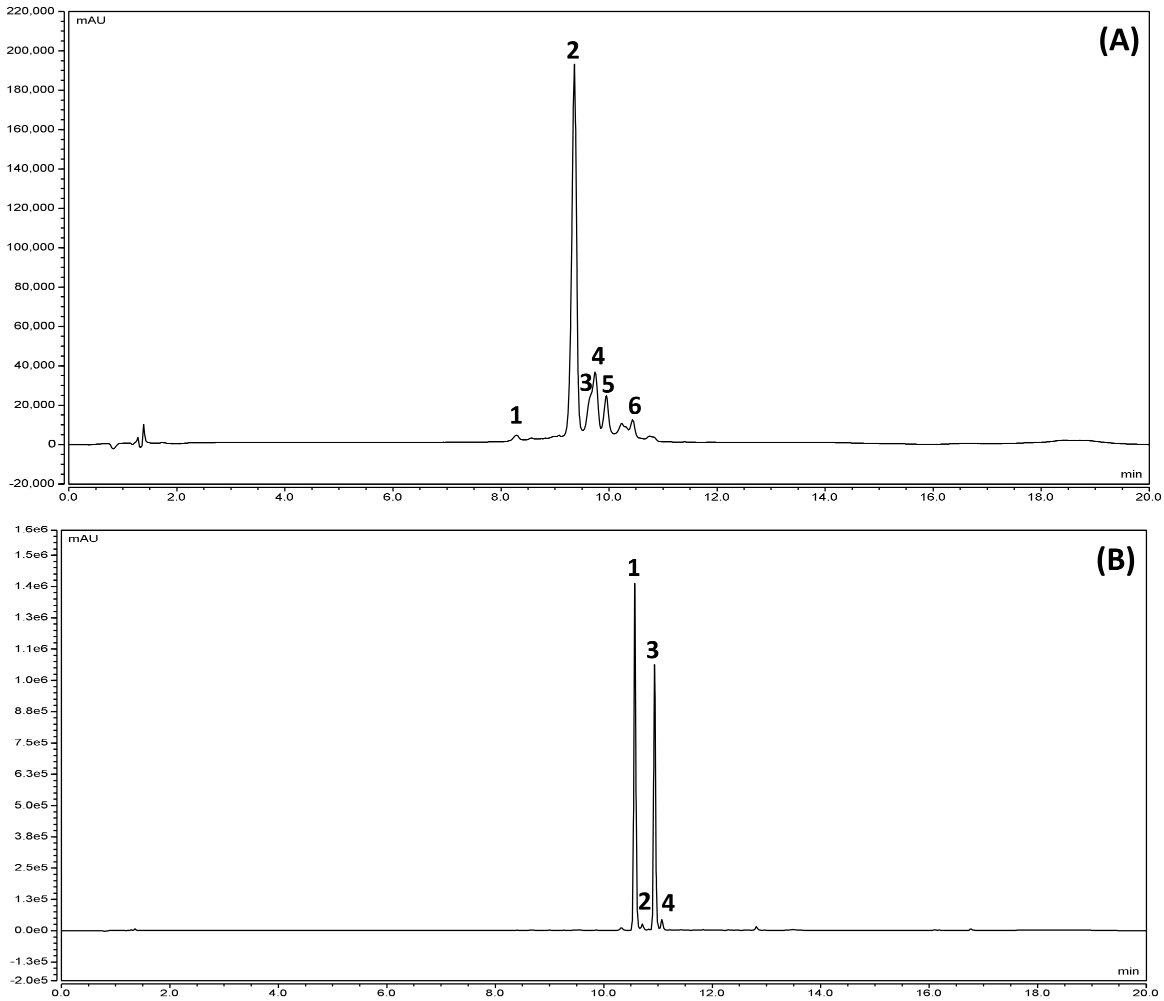

Supplementary Figure S4. UHPLC chromatograms of purple corn cob sample from the highland geographical location. (A): anthocyanin free fraction at 525 nm, (B): bound phenolic fraction at 320 nm.

Phenolic fraction	Peak number	Retention time (min)	λmax (nm)	Compound
Anthocyanin free fraction at 525 nm	1	8.2830	286.64; 537.01	Anthocyanin
	2	9.3580	280.30; 515.99	Anthocyanin
	3	9.6270	279.01; 505.86	Anthocyanin
	4	9.7420	280.04; 517.20	Anthocyanin
	5	9.9520	281.44; 518.69	Anthocyanin
	6	10.437	280.90; 521.16	Anthocyanin
Bound phenolic fraction at 320 nm	1	10.572	309.95	*p*-coumaric acid
	2	10.713	301.03	*p*-coumaric acid derivative
	3	10.937	323.18	Ferulic acid
	4	11.073	317.68	Ferulic acid derivative

L-Valine

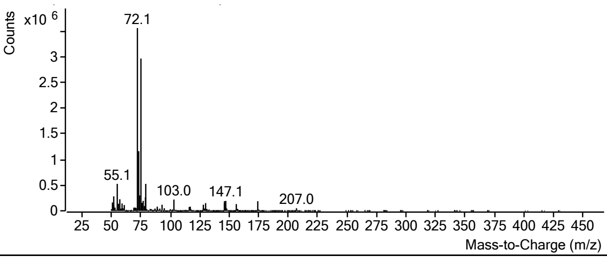

L-Alanine

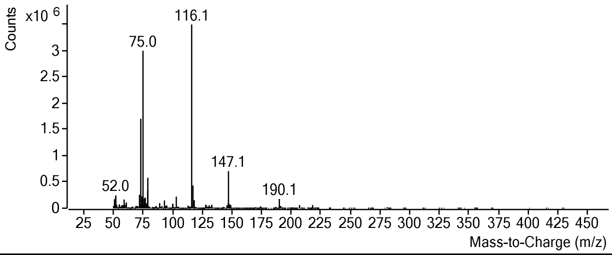

L-serine

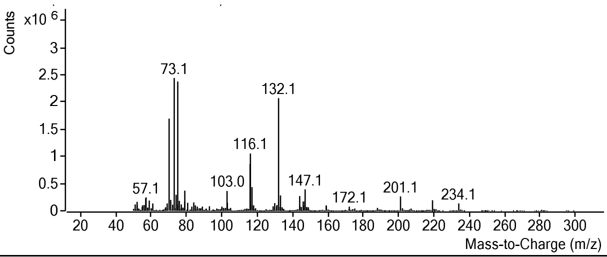

Ethanolamine

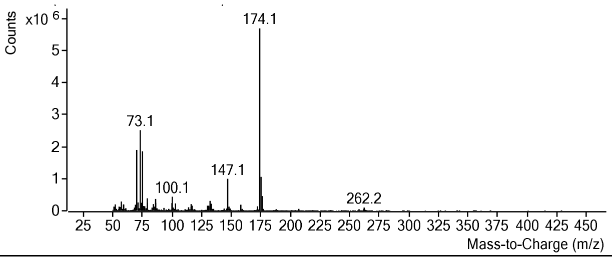

Butanedioic acid
(Succinic acid)

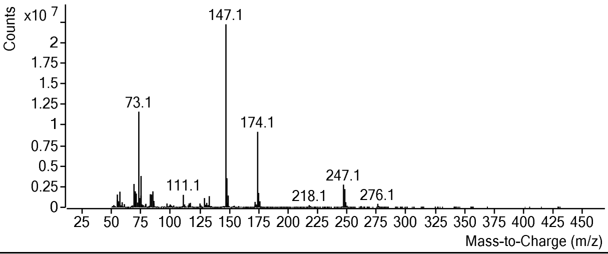

Glyceric acid

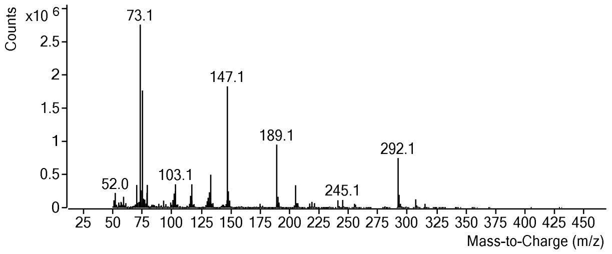

2-Butenedioc acid
(Fumaric acid)

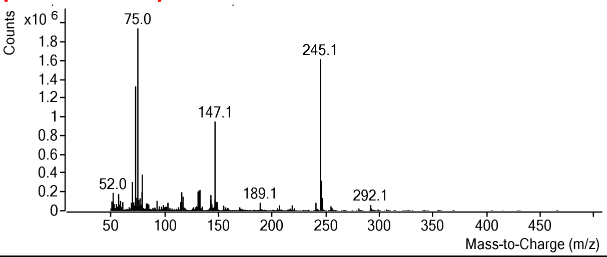

L-5-oxoproline

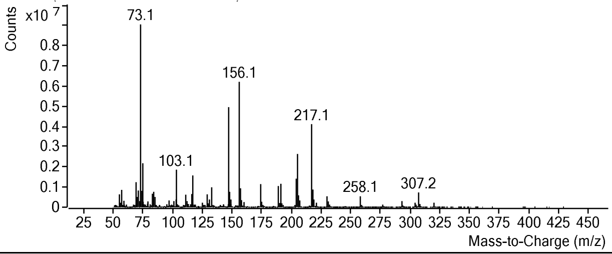

4-Aminobutanoic acid
(GABA)

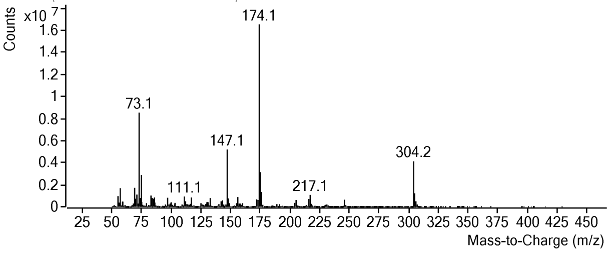

D-Glucitol
(D-sorbitol)

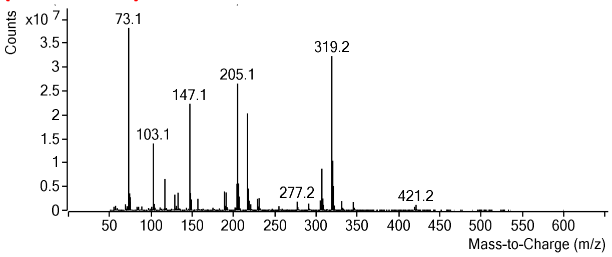

Sucrose

D-Mannose

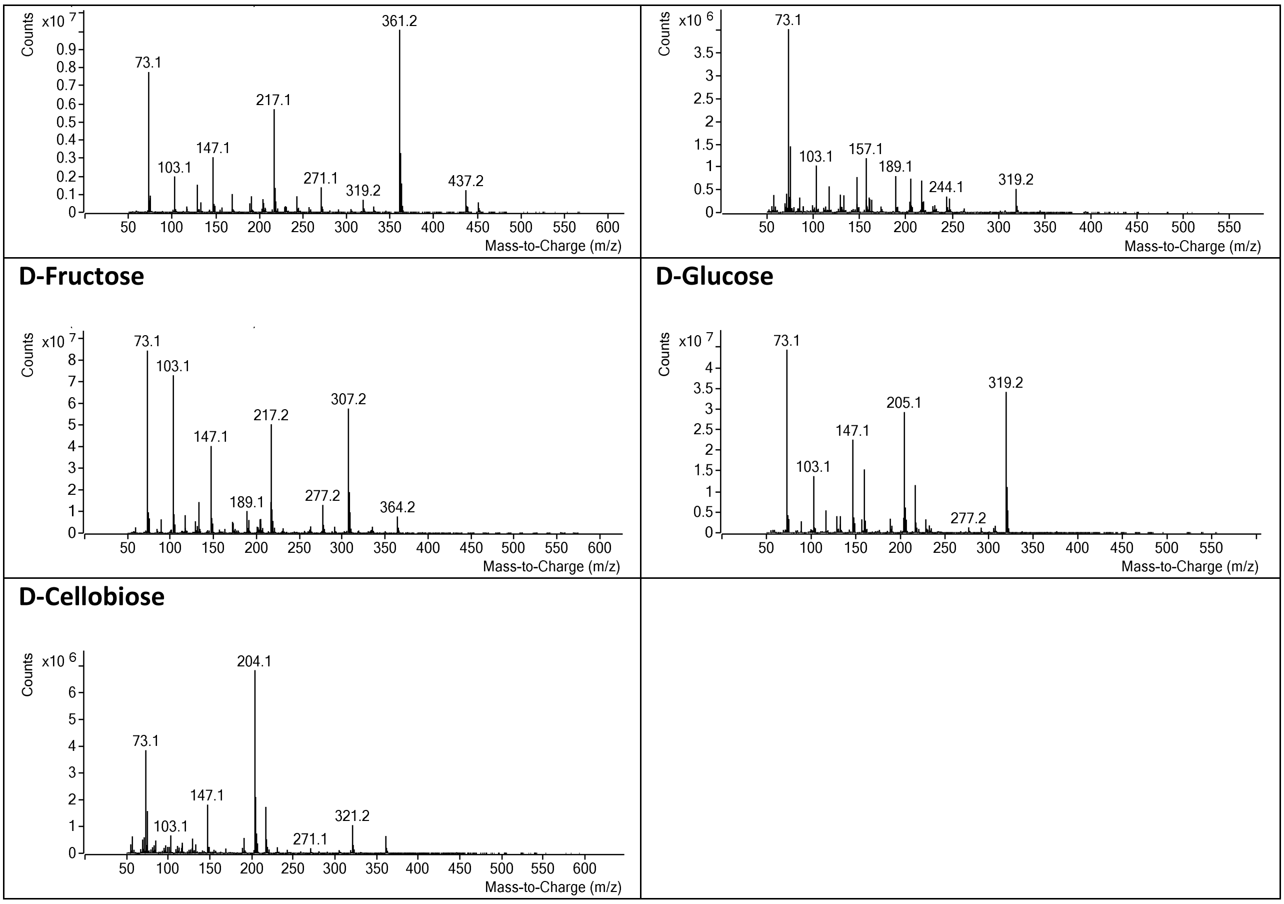

Supplementary Figure S5. Mass spectra of significant polar primary metabolites detected in purple corn kernels by CG-MS.

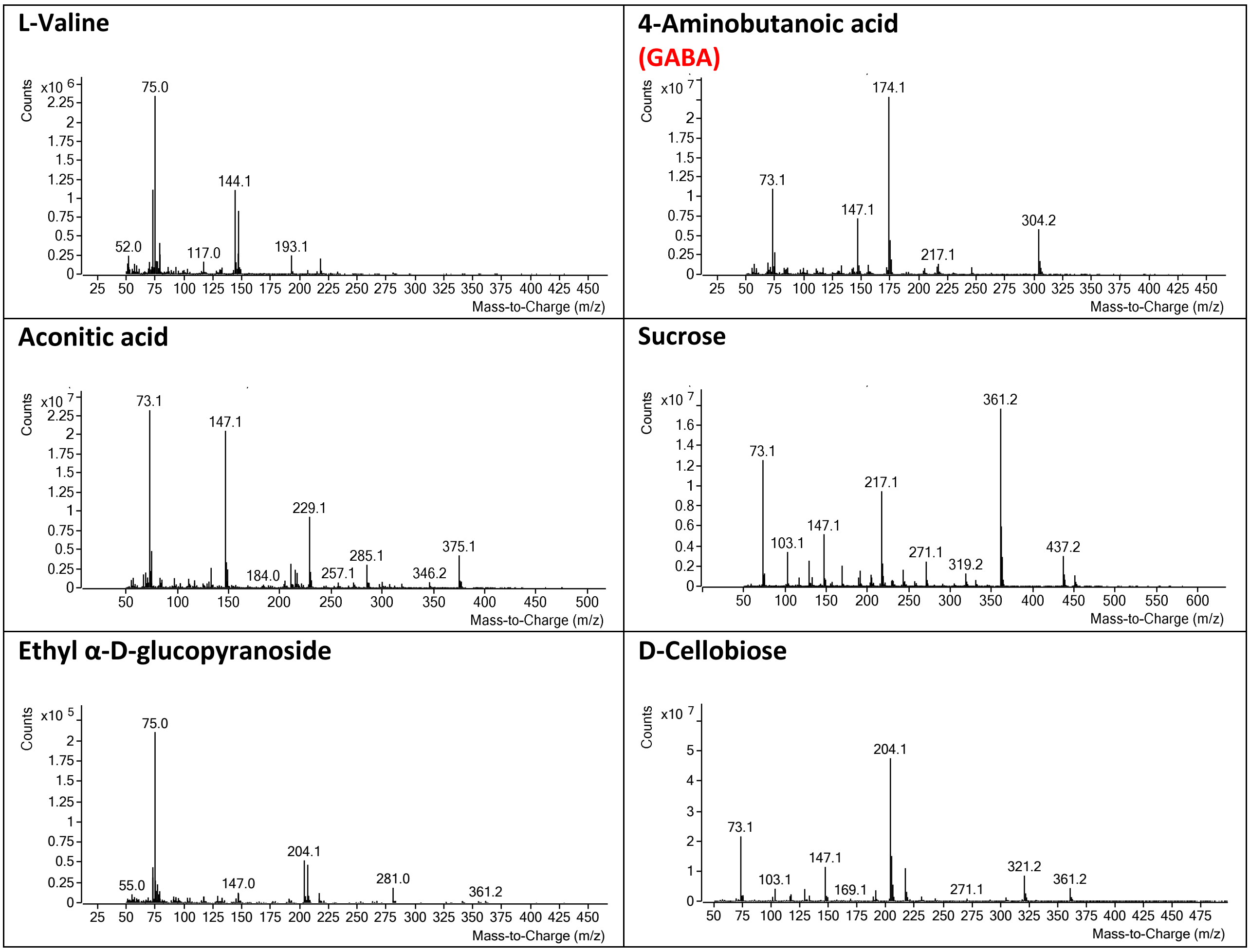

Supplementary Figure S6. Mass spectra of significant polar primary metabolites detected in purple corn cobs by CG-MS.

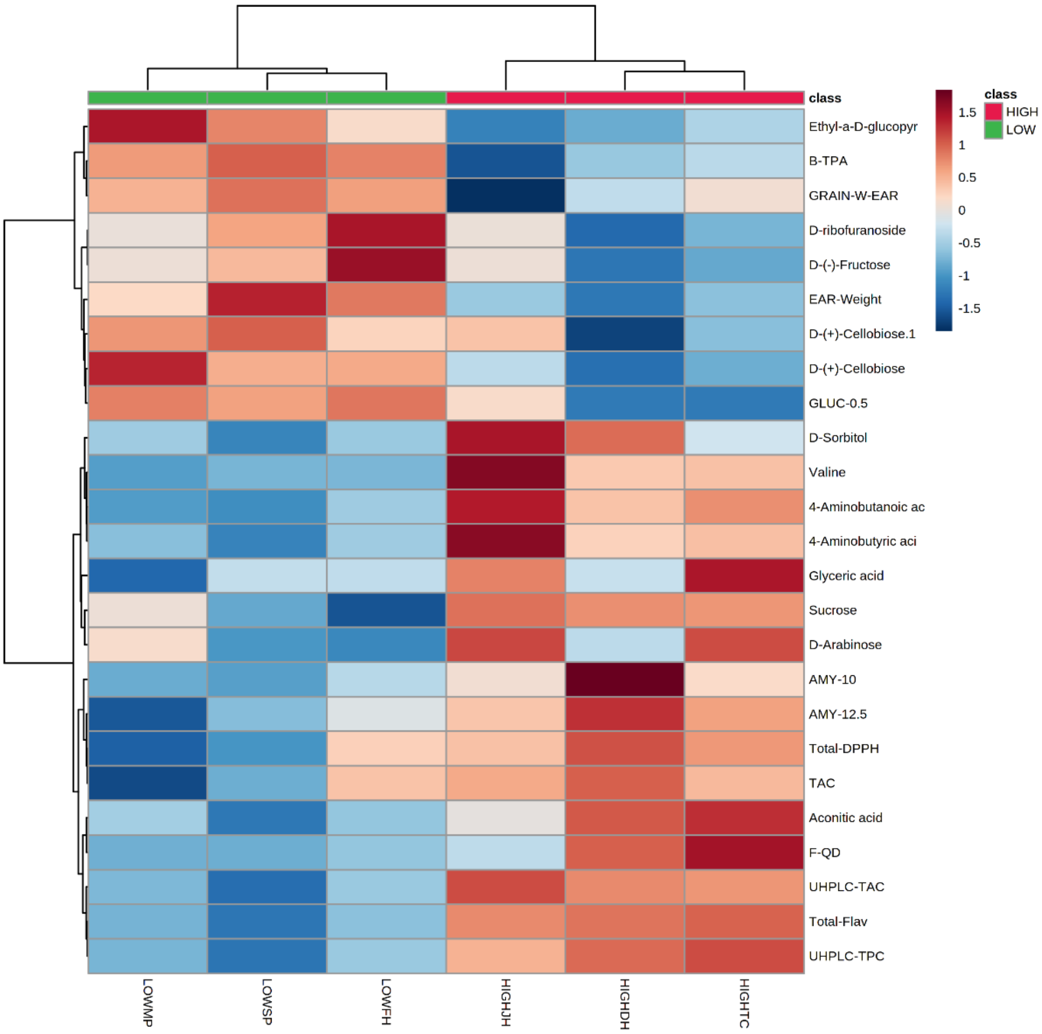

Supplementary Figure S7. Heat map considering the top 25 t-test variables in case of purple corn cob samples grown at two different geographical locations (highland, HIGH and lowland, LOW). Variables in legend: Ethyl-a-D-glucopyr, ethyl-a-D-glucopyranoside; B-TPC, bound total phenolic acid contents; GRAIN-W-EAR, kernel weight per ear; D-ribofuranoside; methyl-a-D-ribofuranoside; GLUCO 0.5, α-glucosidase inhibition (0.5 mg); AMY-10, α-amylase inhibition (10 mg); AMY-12.5, α-amylase inhibition (12.5 mg); Total-DPPH, total DPPH antioxidant capacity; TAC, total monomeric anthocyanin contents; F-QD, free quercetin derivatives; UHPLC-TAC, UHPLC total anthocyanins; Total-flav, total flavonoids; UHPLC- TPC, UHPLC total phenolic compounds.
